# *Histoplasma capsulatum* Not Detected in People with HIV and Systemic Symptoms in Port-au-Prince, Haiti

**DOI:** 10.1177/08892229251365925

**Published:** 2025-10-03

**Authors:** Kathryn M. Dupnik, Nancy Dorvil, Maureen Ward, Vanessa Rivera, Alexandra Apollon, Emelyne Dumont, Omar Sued, Jean W. Pape, Serena P. Koenig

**Affiliations:** ^1^ Department of Medicine, Weill Cornell Medicine, New York, New York, USA.; ^2^ Haitian Group for the Study of Kaposi’s Sarcoma and Opportunistic Infections (GHESKIO), Port-au-Prince, Haiti.; ^3^ HIV, Tuberculosis and Viral Hepatitis Unit, Pan American Health Organization, Washington, District of Columbia, USA.; ^4^ Brigham and Women’s Hospital, Harvard Medical School, Boston, Massachusetts, USA.

**Keywords:** *Histoplasma*, fungal infections, Haiti, HIV, AIDS

## Abstract

When local epidemiology of endemic fungal infections is poorly understood, it is difficult to determine the clinical impact of screening for these pathogens in people living with HIV. We found no *Histoplasma* urine antigen or serum antibody in over 450 symptomatic patients with a new diagnosis of HIV in Port-au-Prince, Haiti. These findings indicate that systematic testing is likely to be low yield in Port-au-Prince, although it is well described in other countries in the region, and may have higher prevalence in rural areas. These findings are relevant to other settings where the prevalence of histoplasmosis is poorly defined, as similar studies could also inform local evidence-based guidelines.

Despite expanded access to antiretroviral therapy, a substantial proportion of people living with HIV (PWH) present with advanced HIV disease. Systemic symptoms are common during advanced HIV, with a broad list of potential etiologies including tuberculosis (TB) and histoplasmosis. Histoplasmosis is on the WHO Fungal Priority Pathogens List as an opportunistic infection that causes invasive disease in PWH^1^ and should be included in the differential diagnosis for people with advanced HIV and pulmonary or systemic symptoms.^2–4^ Histoplasmosis has been identified in Africa, Asia, and the Americas, suggesting that systematic screening of PWH may be indicated globally.^5–7^ The Pan American Health Organization is supporting implementation projects in multiple countries to determine who should be screened for histoplasmosis.^8,9^

Pulmonary histoplasmosis shares many clinical and radiographic similarities with pulmonary TB, and in the right environment, these two infections can be confused with each other. In addition, people with advanced HIV can have coinfection with both *Mycobacterium tuberculosis* and *Histoplasma* when living in an area endemic for both infections.^10^ Rates of co-occurrence of TB in people with histoplasmosis have ranged from 2% to 38% in studies in South and Central America and the Caribbean, as summarized in Caceres et al.^10^ Coinfection is an important consideration for appropriate antimicrobial treatment selection as well as timing of initiation of antiretroviral therapy (ART) for people newly diagnosed with HIV.

Histoplasmosis has been called an “invisible elephant” and “lethal blind spot” in Central and South America.^11–13^ Histoplasmosis also has been described in the Caribbean region,^14,15^ including Martinique,^16,17^ Guadeloupe,^18^ Jamaica,^19,20^ and Trinidad and Tobago.^21,22^ A modeling study that extrapolated data from French Guiana suggested that more PWH in the Caribbean die of histoplasmosis than TB.^12^ An outbreak of histoplasmosis associated with exposure to bat guano during tunnel work was described in the Dominican Republic, which shares the island of Hispaniola with Haiti, although a comprehensive national epidemiological survey has not been completed in the Dominican Republic.^23^ Given the high prevalence of histoplasmosis in other Caribbean nations and the unknown seroprevalence in Haiti, our objective was to determine the prevalence of histoplasmosis in people with new diagnosis of HIV at our center in urban Haiti.

This study was approved by the Institutional Review Boards at GHESKIO (Haitian Group for the Study of Kaposi’s Sarcoma and Opportunistic Infections), Weill Cornell Medicine, and Mass General Brigham. Written informed consent to participate in the original study was obtained from all participants, including permission for samples to be used for future studies. The research conducted was in accordance with the Declaration of Helsinki as revised in 2013.

This study was conducted at GHESKIO, a Haitian nongovernmental organization that provides comprehensive services for HIV and TB in Port-au-Prince, Haiti. A total of 500 adults (≥18 years of age) who presented with TB symptoms (cough, fever, night sweats, and/or weight loss) at HIV diagnosis were enrolled in a study of same-day versus conventional evaluation for active pulmonary TB before starting ART (SDART/TB study) between November 2017 and January 2020.^24^ Exclusion criteria included symptoms consistent with WHO stage 4 neurological disease or WHO “danger signs” of temperature >39°C, pulse >120 beats/min, respiratory rate >30, or inability to walk unaided. All participants received two Xpert MTB/RIF tests and two mycobacterial cultures. Bacteriologically confirmed TB was defined as positive Xpert MTB/RIF or mycobacterial culture. Empirical TB was defined as negative microbiologic tests but high clinical suspicion for TB based on chest X-ray and clinical symptoms. At enrollment, serum and urine samples were collected and frozen at –80°C.

Urine *Histoplasma* antigen was assayed using an enzyme immunoassay (EIA; clarus Histoplasma GM, IMMY, Oklahoma, USA) according to the manufacturer’s instructions for the calibrator cutoff procedure. This EIA is approved for urine antigen testing only. Each EIA plate passed quality control with positive and negative controls and calibrator in the appropriate range of calculated EIA units. Serum *Histoplasma* antibody was assayed using immunodiffusion with the *Histoplasma* reagent set (IMMY) with a 30-min incubation of samples and controls before adding the *Histoplasma* antigen. Each set of samples tested included positive controls. Results were read after both 24 and 48 h of incubation.

For the parent study, 576 people were screened and 500 were enrolled. All enrolled participants lived in the Port-au-Prince metropolitan area. Of the 76 patients who failed screening, 9 had a WHO “danger sign,” and the remainder were excluded for reasons unrelated to disease severity, including failing an ART readiness assessment (*n* = 20), ART-experienced (*n* = 18), declining study participation (*n* = 11), and pregnancy or breastfeeding (*n* = 9).^24^ Of the 500 participants enrolled in the parent study, serum was available for 498 and urine for 460. Of these, the median CD4 count was 276 cells/mm^3^ (interquartile range [IQR]: 128, 426) and 181 (36.3%) had CD4 counts <200 cells/mm^3^ (Table 1). A total of 87 (17.5%) participants were diagnosed with TB, of whom 67 were bacteriologically confirmed. Fifty-five participants had positive GeneXpert testing, while the remainder (*n* = 32) had positive cultures or were treated empirically without microbiological confirmation. A total of 445 (89.4%) people were retained in care at 48 weeks, of whom 15 (3.0%) died. Histoplasmosis was not considered a probable etiology for systemic symptoms or a cause of death in any participant.

**Table 1.** Demographics of Cohort Participants Who Had Urine and Serum Available for *Histoplasma* Testing

Variable	Cohort with sera for testing (*n* = 498)	Cohort with urine for testing (*n* = 460)
Female—no. (%)	234 (47.0)	218 (47.4)
Age (years)—median (IQR)	37 (30, 45)	37 (30, 45)
Cough, fever, and/or night sweats—no. (%)	260 (52.2)	240 (52.2)
Body mass index—median (IQR)	20.7 (18.7, 22.9)	20.7 (18.7, 23.0)
CD4 count category—no. (%)^a^		
<200 cells/mm^3^	181 (36.3)	168 (36.5)
200 to 349 cells/mm^3^	143 (28.7)	129 (28.0)
350 to 499 cells/mm^3^	84 (16.9)	74 (16.1)
≥500 cells/mm^3^	85 (17.1)	84 (18.3)
CD4 count—median (IQR)^a^	276 (128, 426)	276 (136, 431)
Hemoglobin (g/dL)—median (IQR)	11.9 (10.1, 13.4)	11.9 (10.2, 13.4)
C-reactive protein [mg/L—median (IQR)]	3.9 (1.0, 18.7)	3.7 (0.9, 17.4)
Tuberculosis diagnosis—no. (%) (bacteriologically confirmed + empirically treated)	87 (17)	78 (17.0)
*Histoplasma* antigen positive—no./tested (%)	N/A	0/460 (0%)
*Histoplasma* antibody positive—no. (%)	0/498 (0%)	N/A

^a^ Seven people in cohort with sera had missing CD4 data. Five people in cohort with urine had missing CD4 data.

IQR, interquartile range; N/A, not applicable.

All urine specimens were negative for *Histoplasma* antigen, and all sera were negative for *Histoplasma* antibody. In this cohort of patients with at least one TB-like symptom at HIV diagnosis, no patient tested positive for histoplasmosis by urine or serum testing, including 32 people with initial laboratory testing negative for *M. tuberculosis*. This is important because histoplasmosis has been reported as a common etiology for patients with systemic or pulmonary symptoms in other countries in the region, including as a frequent etiology of “smear-negative TB.” These findings indicate that systemic testing for histoplasmosis is likely to be low yield in Port-au-Prince.

*Histoplasma* urine antigen testing is 95% sensitive for histoplasmosis in PWH^25^ and is the screening test recommended by the Neglected Histoplasmosis in Latin America group.^12^ Urine antigen testing sensitivity is lower in people with immune suppression and in isolated pulmonary compared with disseminated histoplasmosis. The product insert for the IMMY *Histoplasma* antigen EIA urine test indicates that a “very low-positive specimen could become negative after storage,” although the time period after which a low-positive urine could become negative was not specified. To substantiate a lack of histoplasmosis in this cohort, we assayed *Histoplasma* serology, which was negative for all samples. We also note that this cohort was followed closely for at least a year, and no patient developed a clinical presentation that was felt to be consistent with histoplasmosis. Our study was limited by not including more laboratory investigations for histoplasmosis, including intradermal testing, serologic testing using complement fixation, and serum antigen testing. These tests could have detected exposure and infection in people who were serum immunodiffusion and urine antigen negative. Other potential contributors for false-negative histoplasmosis test results could be the following: (1) Antigen test could be negative in people without advanced HIV and with high CD4 T cell count. (2) People with low CD4 may be less likely to produce antibodies and could have false-negative *Histoplasma* serologies. It is our hope that by combining assays for serology and antigen we would detect evidence of *Histoplasma* infection if it were present, by antigen for people with low CD4 and by serology for people with higher CD4.

While histoplasmosis is a common cause of morbidity and mortality for people with advanced HIV infection in some countries in the region, our findings lead us to conclude that the prevalence of histoplasmosis in Port-au-Prince is very low. Therefore, according to the Infectious Diseases Society of America guidelines for histoplasmosis, which advocate for universal screening and itraconazole prophylaxis for people with CD4 <150 cells/mm^3^ when the rate of histoplasmosis is over 10 cases/100 patient-years, routine screening is not indicated in our setting.^26^ We hypothesize that Port-au-Prince has isolated or no ecological niches for *Histoplasma* to persist in the environment where people would be exposed to it, although further research is needed to assay for *Histoplasma* in the environment. There are 18 species of bats on Hispaniola, which comprises Haiti and the Dominican Republic, but it is unclear how these bats are currently distributed across Haiti.^27^ Living bats characterized in the greater Port-au-Prince area were collected in the 1930s from homes, caves, and “sleeping in mango trees.”^28^ Histoplasmosis might be more common in rural areas with habitat, environmental conditions, and host animals conducive to *Histoplasma*.^29^ The International Histoplasmosis Advocacy Group issued the Manaus Declaration in 2019, calling for access to rapid testing for all countries in the Americas by 2025.^30^ Expanded access to affordable assays is necessary to describe the regional epidemiology of histoplasmosis.

In conclusion, we detected no histoplasmosis among a cohort of symptomatic patients at HIV diagnosis in Port-au-Prince. These findings indicate that systematic testing is likely to be low yield in Port-au-Prince, although it is well described in other countries in the region, and may have higher prevalence in rural areas. These findings are relevant to other settings where the prevalence of histoplasmosis is poorly defined, as similar studies could also inform local evidence-based guidelines.

## Data Availability

The deidentified dataset of the original cohort was published in *PLOS Medicine*.^24^ Reasonable requests for additional data will be considered by the PI of the parent study (S.P.K.).
